# Supplementary material for: Convergent allostery in ribonucleotide reductase
Source: Nat Commun. 2019 Jun 14;10:2653. doi: 10.1038/s41467-019-10568-4 (PMC6572854; doi:10.1038/s41467-019-10568-4)
Supplement: Supplementary file 1 — Supplementary Information [file 41467_2019_10568_MOESM1_ESM.pdf]

Convergent Allostery in Ribonucleotide Reductase

W. C. Thomas et al.

Supplementary Table 1. Summary of SAXS experiments

Experiment	Type	Figure
80 µM wt holo-NrdE in assay buffer with 1% glycerol	SEC	2a
40 µM wt apo-NrdE in assay buffer with 5% glycerol	SEC	2a
40 µM wt holo-NrdE + 100 µM dATP, 0.5 mM CDP in assay buffer with 1% glycerol	SEC	2a, S2b
4 µM wt holo-NrdE + 0-50 µM dATP, 1 mM CDP in assay buffer with 1% glycerol	Titration	2b, S2a
4 µM wt apo-NrdE + 0-50 µM dATP, 1 mM CDP in assay buffer with 5% glycerol	Titration	2b, S2a
4 µM C382S holo-NrdE + 0-20 µM Fe-NrdF, 50 µM dATP, and 1 mM CDP in assay buffer with 5% glycerol	Titration	2c
4 µM wt holo-NrdE + 0-1 mM dATP, 1 mM CDP in assay buffer with 1% glycerol	Titration	3a
4 µM wt apo-NrdE + 0-1 mM dATP, 1 mM CDP in assay buffer with 5% glycerol	Titration	3a
40 µM wt holo-NrdE + 1 mM ATP, 0.5 mM CDP in assay buffer with 1% glycerol	SEC	S7a
4 µM wt holo-NrdE + 0-15 mM dATP, 50 µM dATP, 1 mM CDP in assay buffer with 1% glycerol	Titration	3b
4 µM wt apo-NrdE + 0-15 mM dATP, 50 µM dATP, 1 mM CDP in assay buffer with 5% glycerol	Titration	3b
4 µM wt holo-NrdE + 0-500 µM TTP, 3 mM ATP in assay buffer with 1% glycerol	Titration	3c
4 µM wt apo-NrdE + 0-500 µM TTP in assay buffer with 5% glycerol	Titration	3c
80 µM wt holo-NrdE + 250 µM TTP, 1 mM ATP in assay buffer with 1% glycerol	SEC	S7b
4 µM wt holo-NrdE + 0-500 µM TTP in assay buffer with 1% glycerol	Titration	3d
40 µM wt holo-NrdE + 100 µM TTP in assay buffer with 1% glycerol	SEC	S2c
80 µM C382S holo-NrdE + 80 µM Mn-NrdF, 1 mM ATP, 250 µM TTP in assay buffer with 5% glycerol	SEC	5a, S13

Assay buffer = 50 mM HEPES pH 7.6, 150 mM NaCl, 15 mM $MgCl_2$, 1 mM TCEP, and 1 or 5% (w/v) glycerol. To prevent changes in nucleotide concentrations, C382S NrdE was used in all experiments where NrdF was added to the mix.

Supplementary Table 2. **SEC-SAXS experimental parameters and data statistics**

	Apo-NrdE	Holo-NrdE	NrdEF + dATP	NrdEF + TTP/ATP
**Beamline parameters**				
Energy (keV)	9.853	9.9496	9.853	9.924
SAXS detector	Pilatus 100K	Pilatus 100K	Pilatus 100K	Pilatus 100K
WAXS detector	Pilatus 100K	Pilatus 100K	Pilatus 100K	Pilatus 100K
q range ($Å^{-1}$)	0.018-0.645	0.008-0.700	0.010-0.645	0.013-0.580
**SEC-SAXS parameters**				
Column	Superdex 200 Increase 3.2/300	Superdex 200 Increase 3.2/300	Superdex 200 Increase 3.2/300	Superdex 200 Increase 10/300
Flow rate (ml min^{-1})	0.1	0.1	0.1	0.5
Loading concentration (μM)	40	80	40	75
Temperature (°C)	4	4	4	4
Total number of exposures	940	635	1003	649
Exposure time (s)	2	2	2	4
Data processing	EFA	EFA	EFA	EFA
**Structural parameters**				
q range ($Å^{-1}$) [$P(r)$]	0.02 - 0.2160	0.008-0.18	n/a*	0.014-0.1860
D_{max} (Å) [$P(r)$]	121	150.42	n/a*	124.06
$I(0)$ [$P(r)$]	762 ± 3	2929 ± 4	n/a*	1439 ± 2
Rg (Å)	30.5 ± 0.2	45.25 ± 0.08	n/a*	42.66 ± 0.05
Porod volume ($Å^3$) [$P(r)$]	143000	251000	n/a*	347000
q range ($Å^{-1}$) [Guinier)]	0.022-0.044	0.010-0.028	n/a*	0.014-0.029
$I(0)$ [Guinier]	761 ± 2	2907 ± 4	~10,000*	1436 ± 2
Rg (Å) [Guinier]	29.5 ± 0.1	44.0 ± 0.1	~100*	42.8 ± 0.1

* The NrdE filament is too large for conventional Guinier and $P(r)$ analyses within the available q-range. Lower estimates for $I(0)$ and R_g are provided.

Supplementary Table 3. Cryo-EM data collection, refinement, and validation statistics

	#1 NrdEF<br>(EMDB-9272)<br>(PDB 6MW3)	#2 NrdE<br>(EMDB-9293)<br>(PDB 6MYX)
**Data collection and processing**		
Magnification	130,000×	36,000x
Voltage (kV)	200	200
Electron exposure (e^- $Å^{-2}$)	32 to 47	20.0
Defocus range (μm)	0.8 to 2.9μm (95%)	1.0 to 3.0 μm
Pixel size (Å)	1.05	1.505
Symmetry imposed	None	Helix: (74.24 Å, -81.28°),<br>(37.12 Å, 139.36°)
Initial particle images (no.)	126,224	85,532
Final particle images (no.)	126,224	85,532
Map resolution (Å)<br>FSC threshold	4.65<br>(0.143)	6*
Map resolution range (Å)	4.3-10.6 (95%)	4.6-9.0 (95%)
**Refinement**		
Initial model used (PDB code)	PDB 6CGL & 6MT9	NrdEF structure
Model resolution (Å)<br>FSC threshold	4.8<br>(0.5)	6.5<br>(0.5)
Map sharpening *B* factor ($Å^2$)	-155	-100
Model composition<br>Non-hydrogen atoms (per ASU)<br>Protein residues (per ASU)<br>Ligands	 11,023<br>1,382<br>dATP	 22,008<br>2,732<br>dATP
B factors ($Å^2$)<br>Protein<br>Ligand	 122.7<br>103.1	 214.01<br>161.61
R.m.s. deviations<br>Bond lengths (Å)<br>Bond angles (°)	 0.006<br>1.072	 0.005<br>0.757
Validation<br>MolProbity score<br>Clashscore<br>Poor rotamers (%)	 1.73<br>4.85<br>0.00	 2.21<br>9.6<br>3.06
Ramachandran plot<br>Favored (%)<br>Allowed (%)<br>Disallowed (%)	 92.20<br>7.80<br>0.00	 95.13<br>4.87<br>0.00

*According to the standard FSC=0.143 criterion, our NrdE map is 4.8 Å resolution. However, according to the features of the map and our map-model FSC, we believe the true resolution of the NrdE map is closer to 6 Å.

Supplementary Table 4. Diffraction data and model refinement statistics for *B. subtilis* NrdE protein crystals

**Data Collection**[a]	Disulfide-trapped NrdE[b] (mainly oxidized) (PDB 6MT9)	X-ray-reduced NrdE[b] (partially reduced) (PDB 6MVE)	NrdE with empty M-site[c] (PDB 6MV9)
Space group	$P4_32_12$	$P4_32_12$	$P2_12_12_1$
Unit cell (Å)	a = b = 126.34, c = 125.44 α = β = γ = 90.0	a = b = 126.41, c = 125.49 α = β = γ = 90.0	a = 120.26; b = 126.40, c = 128.31 α = β = γ = 90.0
Wavelength (Å)	0.9775	0.9775	0.9775
Resolution range (Å)	16.00-2.50 (2.60-2.50)	15.99-2.55 (2.66-2.55)	19.98-2.95 (3.07-2.95)
Total observations	601897 (69514)	439944 (54618)	149881 (17349)
Total unique observations	35371 (3958)	33640 (4072)	41464 (4649)
R_{merge}	0.175 (1.445)	0.162 (1.500)	0.180 (0.763)
R_{pim}	0.041 (0.333)	0.046 (0.424)	0.108 (0.452)
$\langle I/\sigma(I) \rangle$	14.7 (2.2)	11.8 (1.9)	5.4 (1.6)
$CC_{1/2}$	0.998 (0.734)	0.998 (0.745)	0.978 (0.441)
Completeness (%)	99.2 (99.9)	99.6 (100.0)	99.2 (99.8)
Multiplicity	17.0 (17.6)	13.1 (13.4)	3.6 (3.7)
**Refinement Statistics**			
Resolution range (Å)	2.50-16.00	2.55-15.99	2.95-19.98
Reflections (total)	35313	33577	41404
Reflections (test)	2778	2642	950
Total atoms refined	5690	5621	10896
R_{work} / R_{free}	0.178/0.218	0.175/0.218	0.207/0.239
RMSD of bond lengths (Å)/ angles (°)	0.006/0.919	0.008/1.070	0.003/0.619
Ramachandran plot favored/allowed (%)	97.9/2.1	97.3/2.7	96.1/3.9
Mean B value for all atoms ($Å^2$)	48.0	54.0	50.0

[a] Data collection values in parentheses refer to the high-resolution shell; [b] Crystal was grown with GDP substrate in the crystallization solution; [c] Crystal was grown with CDP substrate in the crystallization solution.

Supplementary Table 5. Estimated σ levels corresponding to the contouring thresholds of cryo-EM maps shown in figures

Figure panel	Map	Final map threshold	σ level	Corresp. half-map threshold	σ level
2d	NrdE	4.45	2.9	0.35	4.5
2e	NrdEF (α)	1.14	2.4	0.348	2.9
2e	NrdEF (β)	0.45	0.93	0.238	2.0
2f	NrdEF	1.18	2.4	0.355	3.0
2g	difference	7.73	4.8	n/a	n/a
2h	difference	12.6	7.8	n/a	n/a
S6a	NrdE	4.24	2.8	0.35	4.5
S6b	NrdEF (α)	1.44	3.0	0.45	3.8
S6b	NrdEF (β)	0.45	0.93	0.238	2.0
S6c	NrdEF	1.44	3.0	0.45	3.8

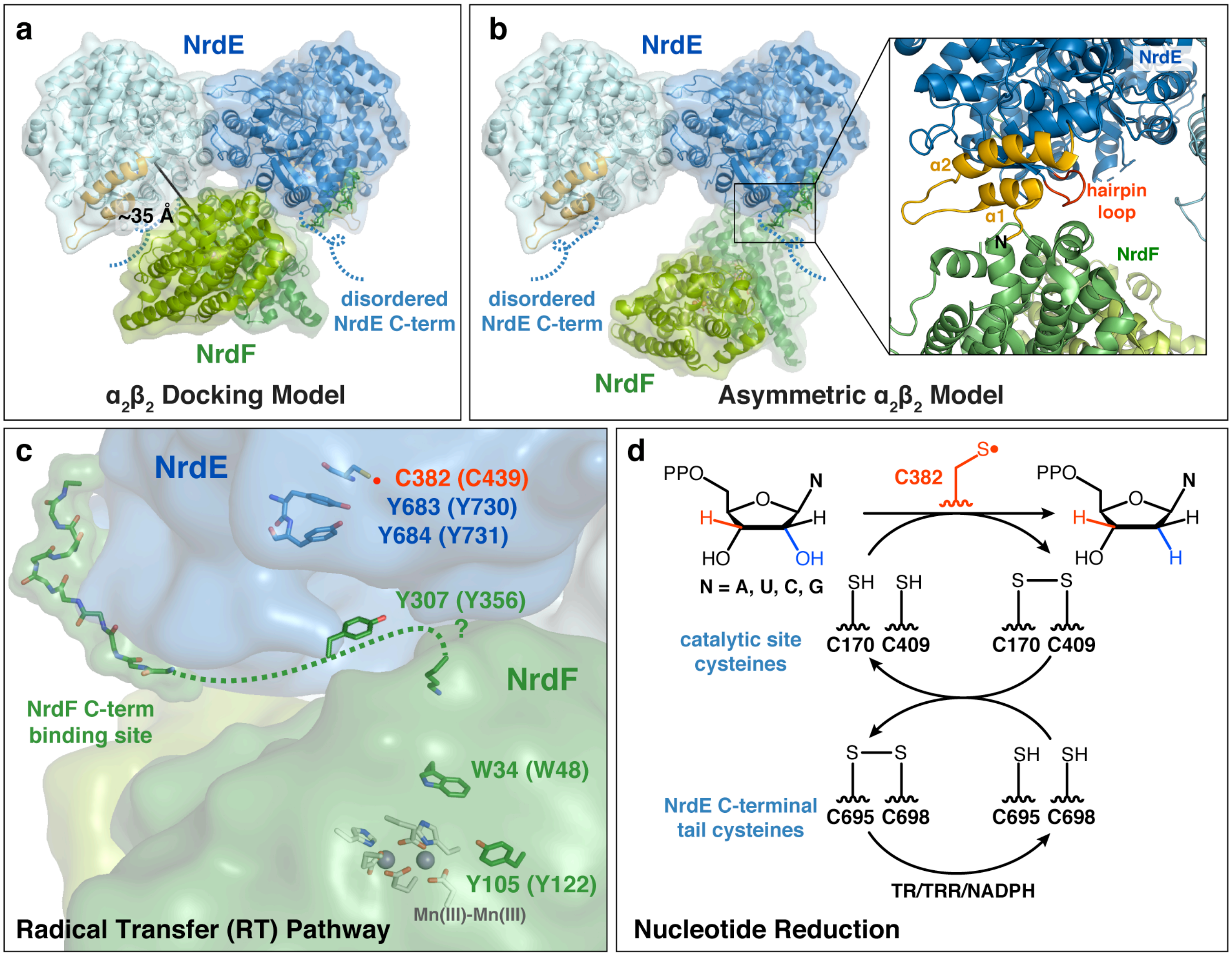

Supplementary Figure 1. Current structural and mechanistic models of class I RNRs and the roles of the C-termini. (a) In class I RNRs, a compact $\alpha_2\beta_2$ configuration is thought to form for radical transfer (RT). Biochemical and low-resolution structural studies on class Ia RNRs support this docking model[1–4]. **(b)** A crystal structure of the *S. typhimurium* class Ib RNR depicts NrdF bound asymmetrically to a single monomer of the NrdE S-dimer (PDB: 2BQ1)[5], forming an interface at the partial ATP-cone (orange cartoon in inset) and a β-hairpin loop (red cartoon in inset). Although this conformation would not be capable of RT, it was proposed to represent a conformation needed for the re-reduction of the catalytic site by the disordered α C-terminus (blue dotted line). **(c)** For each turnover, the catalytic thiyl radical (C382, red) is generated by reversible long-range proton-coupled electron transfer (PCET) over a specific pathway[1]: Y105•/[W34]/Y307 in β to Y684/Y683/C382 in α (*E. coli* Ia numbering shown in parentheses). Brackets indicate that the direct involvement of W34 has not yet been demonstrated. The β C-terminus binds α for complex formation. This tail also contains Y307, the residue that is responsible for RT across the subunit interface, but the residue's location is unknown. This region of the β C-terminus is disordered in all RNR structures (dotted green line). Here, the RT pathway is oriented according to the docking model in (a). **(d)** Once the cysteine radical (red) is generated, nucleotide reduction proceeds using two additional catalytic-site cysteines as reducing equivalents. Two cysteines on the α C-terminus re-reduce the catalytic site and are ultimately reduced by thioredoxin (TR), thioredoxin reductase (TRR), and NADPH.

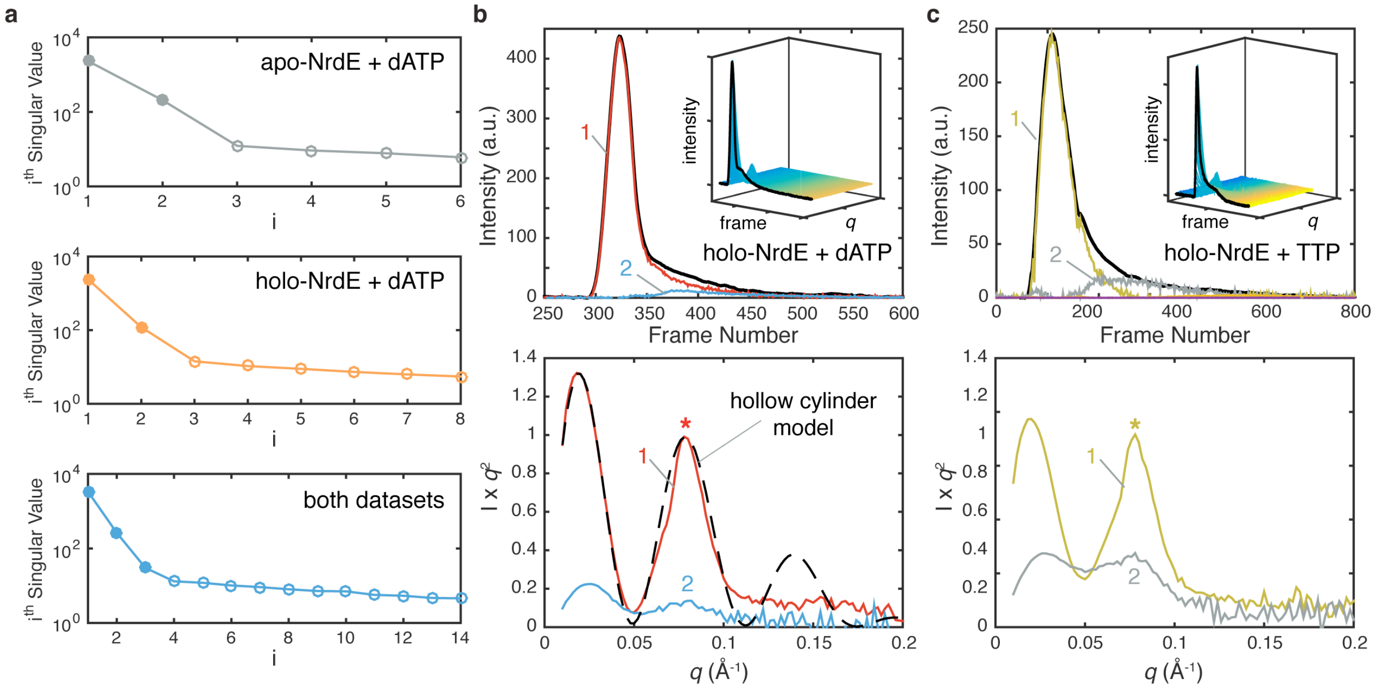

Supplementary Figure 2. SAXS analyses of dATP- and TTP-induced formation of an extended NrdE oligomer. (a) Singular value decomposition (SVD) was performed on dATP titrations shown in Fig. 2b for apo-NrdE (*top*), holo-NrdE (*middle*), and both datasets combined (*bottom*). Significant singular values are shown as closed circles. Apo- and holo-NrdE each have two significant singular values, whereas the combined dataset has three, indicating that the final state of the dATP titration is the same for both samples. **(b)** SEC-SAXS was performed on holo-NrdE with 100 µM dATP (*top, inset*). Evolving factor analysis (EFA) of the dataset (overall elution shown as black curve) yielded two scattering components with the major species (with R_g ~100 Å) eluting first (*top*, red) and scattering features (*bottom*, red) that can be described by a hollow cylinder model with inner radius of 35.2 Å, outer radius of 62 Å, and length of 950 Å (black dashed line). SAXS profiles are shown in Kratky representation to emphasize mid-q features, such as the prominent second peak (star). **(c)** SEC-SAXS was performed on holo-NrdE with 100 µM TTP in the absence of ATP (*top, inset*), and 4 sequentially eluting species were separated using EFA. The major component elutes first (*top*, yellow) and has a similar size (R_g ~100 Å) and shape to the dATP-induced oligomer, including a prominent second peak (*bottom*, star). Components 3 and 4 are not visible in the elution as they are minor contributions. The secondary peak of the extended NrdE oligomer is visible even in the raw SEC-SAXS data (*insets* of (b) and (c)).

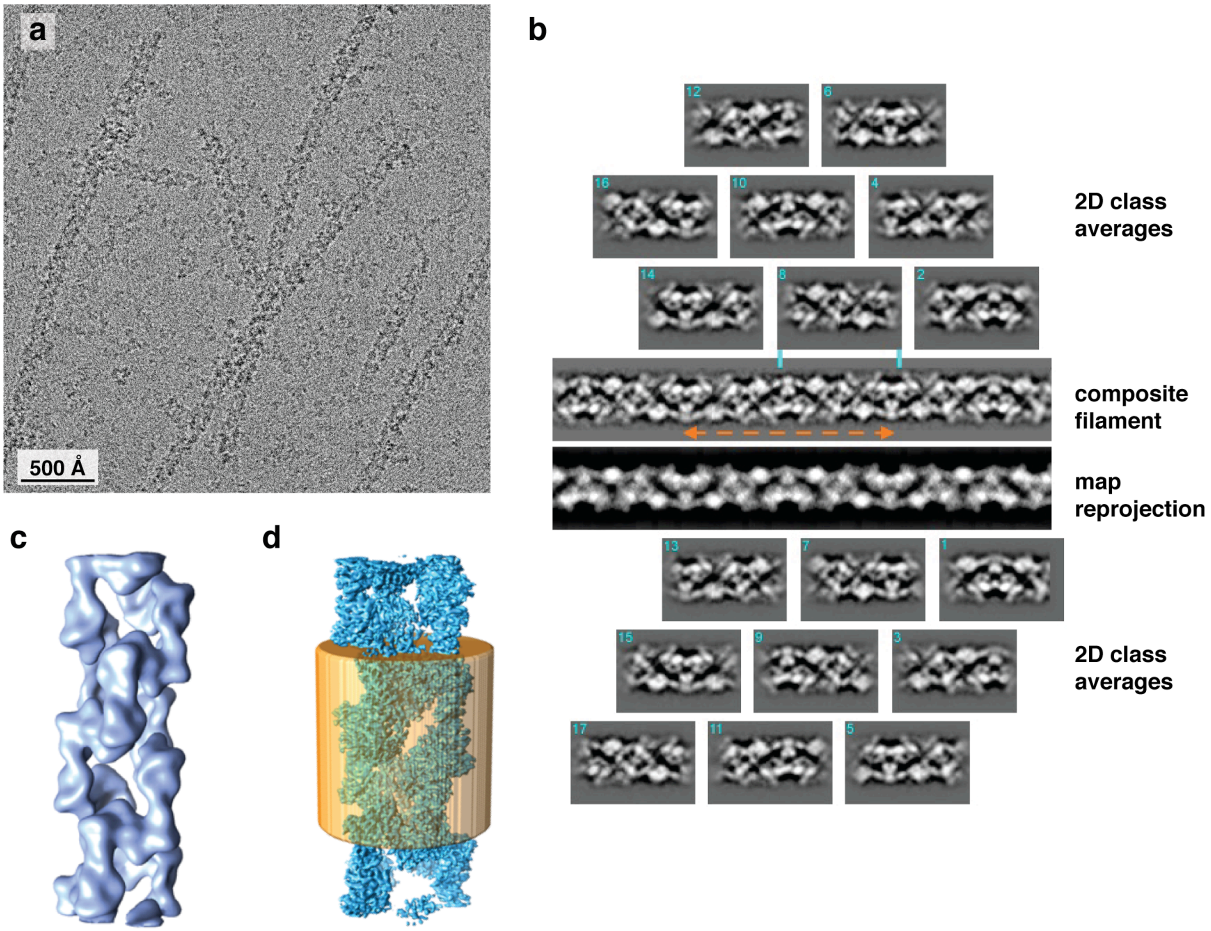

Supplementary Figure 3. Cryo-EM analysis of the dATP-induced NrdE filaments. (a) A representative cryo-EM micrograph of dATP-induced NrdE filaments. **(b)** Here, 17 2D class-average frames are shown vertically aligned to the final composite filament in the middle, with its corresponding model reprojection displayed beneath (low-pass filtered to 15 Å). Measurement of the repeating pattern in the composite filament (orange dashed line) and known dimensions of the NrdE structure provided an initial estimate for the helical parameters. A helical reconstruction test quickly converged to yield initial parameters for subsequent reconstruction and refinement. **(c)** Using the initial model as a reference, a projection-matching algorithm was used in aligning all class averages and subsequent 3D reconstruction-refinement. **(d)** After each refinement-reconstruction iteration, a local search around helical parameters was performed to identify the maximum density cross-correlation within a cylindrical mask in the middle of the double-helix map.

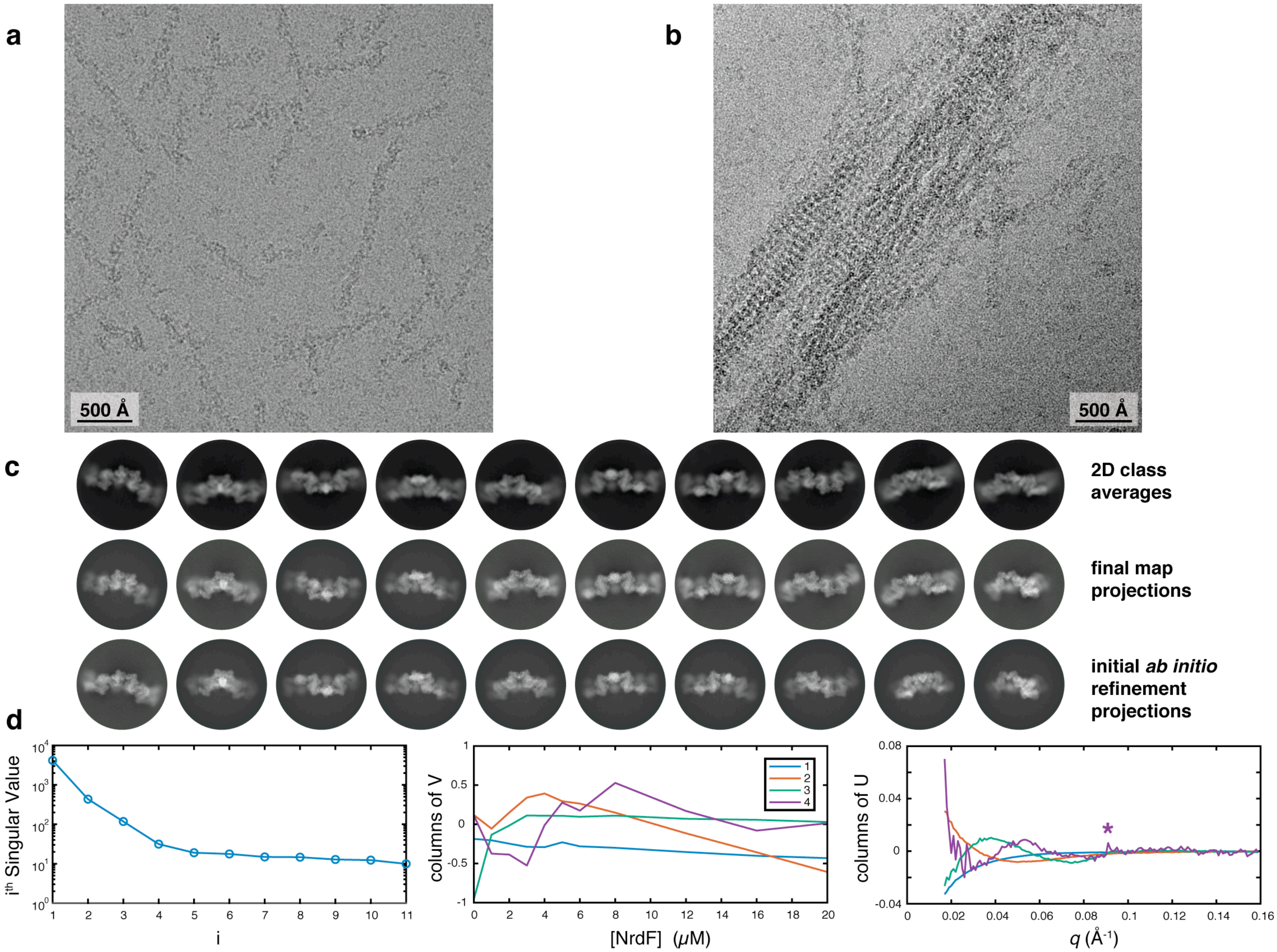

Supplementary Figure 4. Cryo-EM and SAXS analyses of the dATP-induced NrdEF filaments. (a) A representative cryo-EM micrograph of the dATP-induced NrdEF filament. **(b)** The NrdEF filament displayed a tendency to self-associate. Crystalline-like bundles of NrdEF filaments were occasionally but reproducibly observed in micrographs. **(c)** Representative 2D class-averages (top), projections of the final map (middle), and projections of the *ab initio* refinement (bottom) illustrate the agreement between the raw data and 3D reconstruction. **(d)** SVD analysis of SAXS titration data shown in main-text Figure 2c shows that there are four significant species (left panel). The concentration profile of the third SVD component (green, middle panel) drops rapidly from a positive value to roughly zero within the first two titration points. This component disappears if we omit the first scattering profile (0 μM NrdF) from SVD, and thus it represents the change in scattering associated with rapid loss of the NrdE double-helix and formation of the NrdEF filament with the addition of NrdF. The concentration profile of the second SVD component (red, middle panel) has an inflection point around 4 μM NrdF, the presumed saturation point (1:1 subunit stoichiometry), and continues to change linearly with increasing [NrdF]. This component thus represents the change in scattering associated with the buildup of excess NrdF. A unique feature of this subunit titration is the presence of a fourth component associated with a small spike at $q = 0.092$ Å^{-1} (purple, right panel). A peak at this position is consistent with the association of NrdF to the NrdE filament, while the sharpness of this reproducible feature is reminiscent of fiber diffraction. The appearance of fiber diffraction is consistent with the fact that we observed crystalline bundles by EM (as shown in panel b).

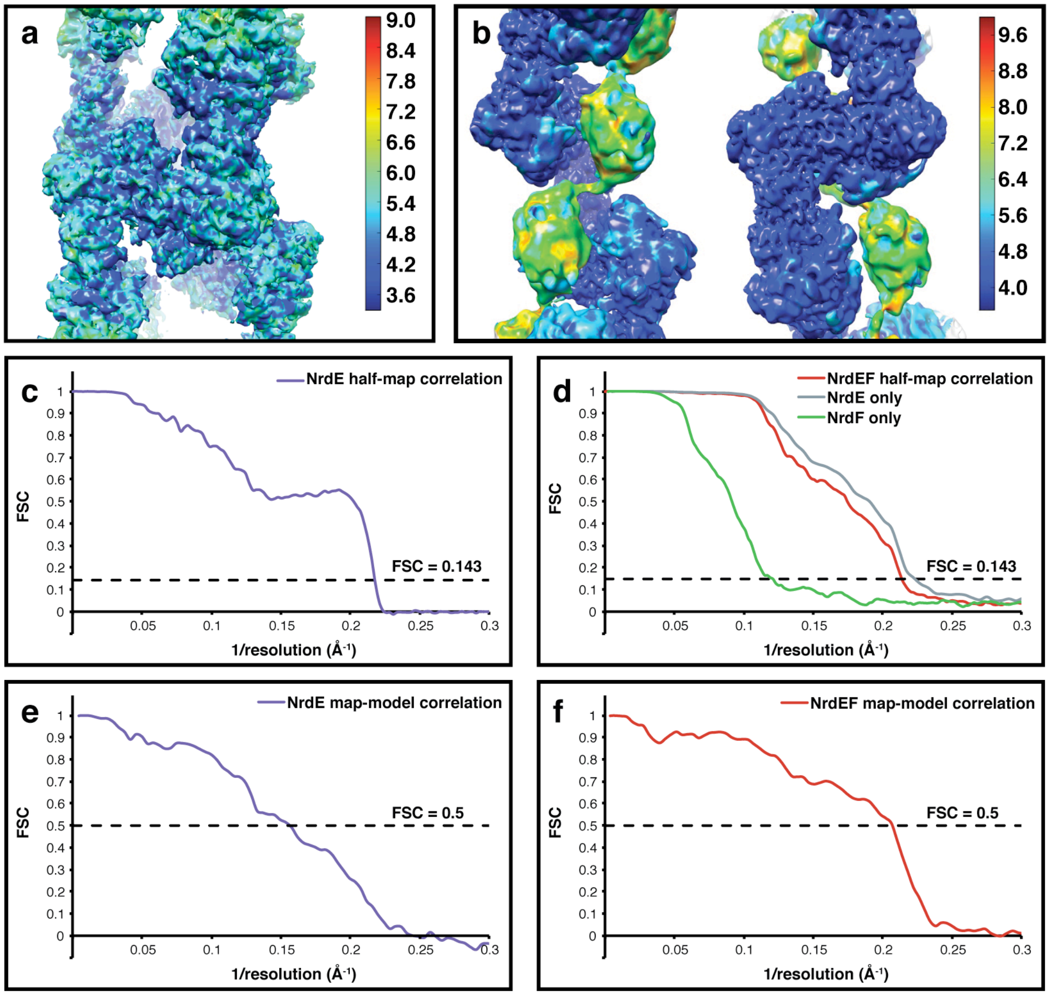

Supplementary Figure 5. Estimation of map and model resolution. Local resolution maps of the **(a)** NrdE filament and **(b)** NrdEF filament, computed in ResMap[6]. The NrdE component of the NrdEF map has much higher resolution features than the NrdF core. **(c)** The Fourier shell correlation (FSC) of independent half maps of the NrdE filament crosses 0.143 at 4.8 Å. However, according to the features of the map and our map-model FSC, we believe the true resolution of the NrdE map to be closer to 6 Å. **(d)** FSC curves of independent half maps of the NrdEF filament (red), density assigned to NrdE in the NrdEF filament (blue), and density assigned to NrdF in the NrdEF filament (green). FSC = 0.143 at 4.4 Å for NrdE alone (blue), 8.2 Å for NrdF alone (green), and 4.65 Å for the total filament (green). **(e)** The map-model FSC calculated for the NrdE filament crosses 0.5 at 6.5 Å. **(f)** The map-model FSC calculated for the NrdEF filament cross 0.5 at 4.8 Å.

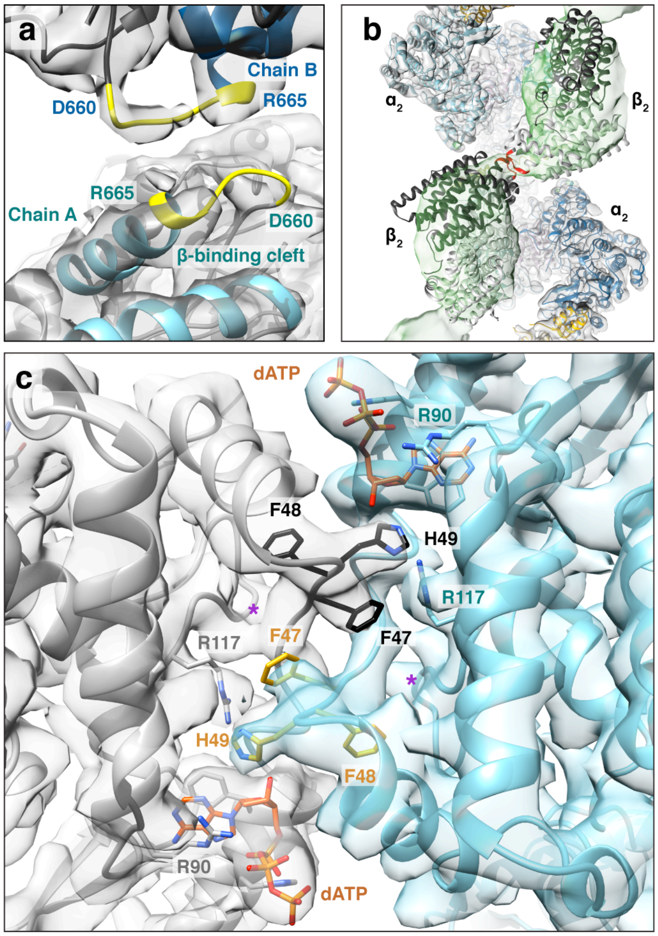

Supplementary Figure 6. Interfaces observed in cryo-EM structures of dATP-induced filaments. (a) The NrdE filament forms the double-helix interface (yellow cartoon) at the β-tail-binding cleft (blue cartoon), which helps explain dissociation of the double-helix upon addition of NrdF. EM density is shown in grey (threshold = 4.24). **(b)** Although density for the NrdF core is weak in our NrdEF cryo-EM map, it has the appropriate size and shape to fit one β_2. Simple rigid-body fitting of the *B. subtilis* NrdF crystal structure (PDB: 4DR0)[7] (black and white) into its density (green, threshold = 0.45) shows that adjacent NrdF dimers would be in close proximity when confined within the helical interior of the NrdEF filament. NrdE density of the NrdEF map is shown in the background (grey, threshold = 1.14). **(c)** The I-dimer interface is formed by interlocking loops (residues 45-50, gold and black) from both chains. At the center of this loop, side-chain density for F47 and H49 is observed extending across the interface. Individual chain density of the NrdEF map is shown here in grey and cyan (threshold = 1.44). The M-site (purple star) is empty.

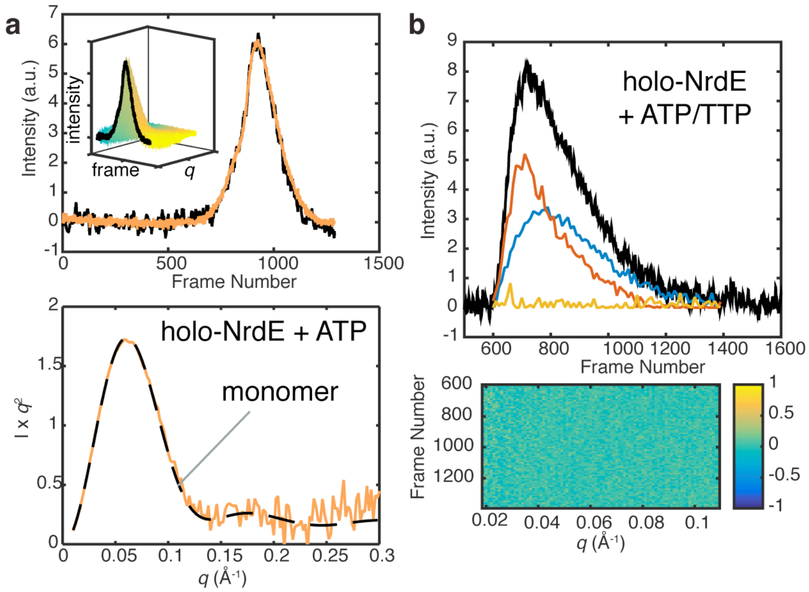

Supplementary Figure 7. SAXS experiments reveal that ATP disrupts the I-dimer interface and specificity effectors induce S-dimer formation. (a) SEC-SAXS was performed on holo-NrdE with 1 mM ATP (*top, inset*). EFA of the dataset (overall elution shown as black curve) yielded a single scattering component (*top*, orange) with scattering features (*bottom*, orange) that are well described by the crystal structure of monomeric NrdE (black dash, PDB: 6CGM)[8]. **(b)** SEC-SAXS of NrdE was performed under conditions with holo-NrdE + 1 mM ATP and 250 μM TTP. The full dataset was fit to a linear sum of the theoretical scattering of the I-dimer (PDB: 6CGL)[8], S-dimer (this work, PDB: 6MT9), and monomer (PDB: 6CGM) using the program OLIGOMER[9,10]. The top panel shows the scattering contribution of S-dimer (red), I-dimer (yellow), and monomer (blue) overlaid with the low-*q* intensity (black) for the SEC elution. NrdE was found to be best explained as a mixture of S-dimer and monomer with no I-dimer component. The co-elution of these species indicates that they are able to rapidly exchange. The bottom panel shows a map of the residuals of the OLIGOMER fits as a function of frame number and *q*. The residuals are largely zero and have no *q*- or frame-dependence.

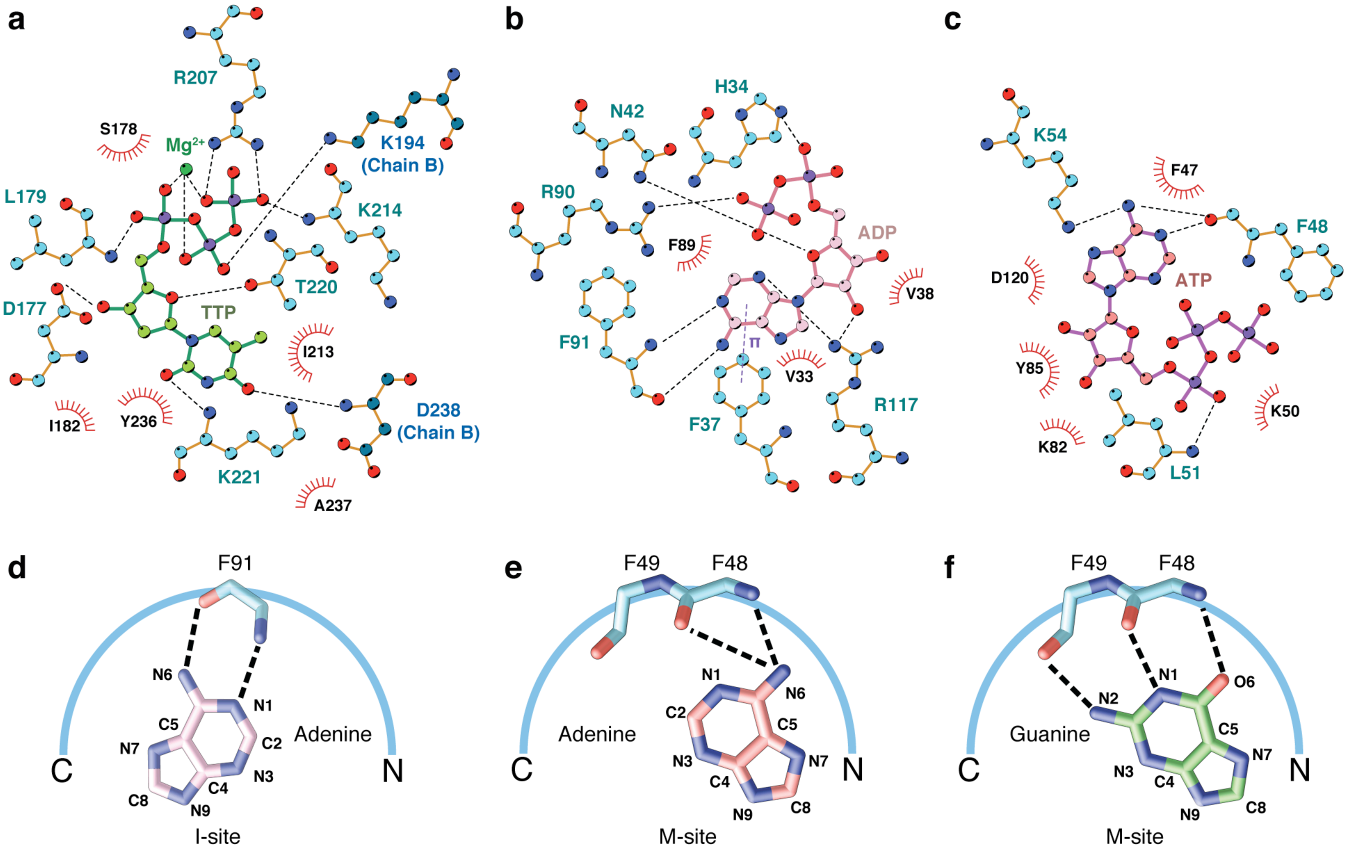

Supplementary Figure 8. Protein-nucleotide interaction diagrams of crystal structures. *Top row:* Ligand-interaction diagrams for our 2.50-Å NrdE S-dimer structure. Polar interactions are shown as dotted lines and hydrophobic contacts are shown as red arcs with radiating spokes. **(a)** Residues from both chains of the S-dimer interact with TTP (carbon atoms shown in green) at the S-site. **(b)** ADP (carbon atoms shown in pink) bound to the I-site. **(c)** ATP (carbon atoms shown in salmon) bound to the M-site. Unless otherwise noted, carbons, oxygens, nitrogens, and Mg^{2+} are shown in light blue, red, dark blue, and dark green, respectively. *Bottom row:* Backbone-nucleobase interactions at the I- and M-sites. **(d)** In all three of our structures as well as structures with dAMP bound[8], the adenine ring of (d)AxP binds the I-site via a reverse adenine-binding interaction using the backbone of residue F91. Here, "reverse" refers to the motif having a C- to N-terminus directionality[11]. **(e)** In our 2.50- and 2.55-Å structures, F48 of the M-site forms a backbone interaction with the adenine ring of ATP, but the positioning prevents interaction of the N1 group with the M-site, as N1 cannot act as an H-bond donor to the backbone carbonyl oxygen of F48. **(f)** Although it is not supported by the electron density in our structures, modeling a guanosine nucleotide in the M-site in the same orientation as ATP reveals three favorable binding interactions. Notably, the N1 group could form an H-bond with the backbone carbonyl of F48.

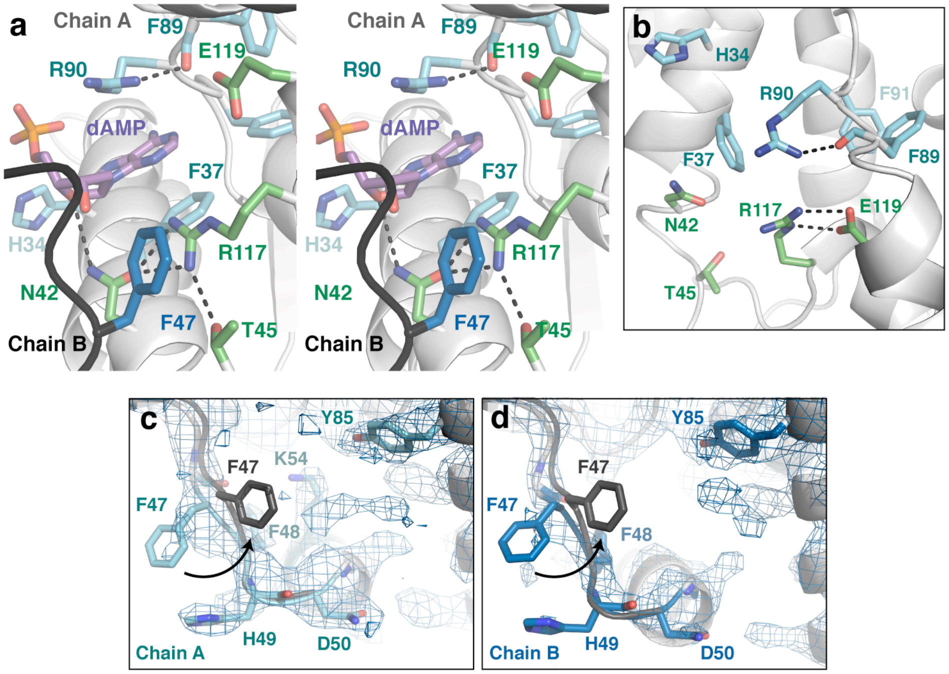

Supplementary Figure 9. The roles of F47 at the I- and M-sites. (a) A stereoview of the I-site in the crystal structure of the dAMP-bound NrdE I-dimer (PDB: 6CGL)[8] reveals that the F47 phenyl ring of one chain (B) is positioned to form a π-cation interaction with R117 of the opposing chain (A). This interaction allows the F47-loop from opposing chains to interlock and form an I-dimer interface. **(b)** In the apo-NrdE structure (PDB: 6CGM)[8], R117 interacts with E119 rather than N42 and T45, thus mimicking the ribonucleotide-bound conformation as seen in Figure 4e. (**c-d**) $2F_o$-F_c electron density map for our 2.95-Å NrdE S-dimer structure obtained with CDP in the crystallization condition is shown as a blue mesh contoured at 1σ. No convincing electron density for a nucleotide is found adjacent to the F47-loop in either (**c**) chain A (cyan) or (**d**) chain B (blue). Consistent with having empty M-sites, the F47-loop is slightly disordered, particularly in chain B, but there is a clear difference in both the side-chain and backbone positions when compared to the F47-loop of our 2.50-Å structure (black cartoon overlay). In our 2.50-Å structure, ATP is bound in the M-site, causing F47 to swing inward (arrow). This is expected to disfavor I-dimer formation.

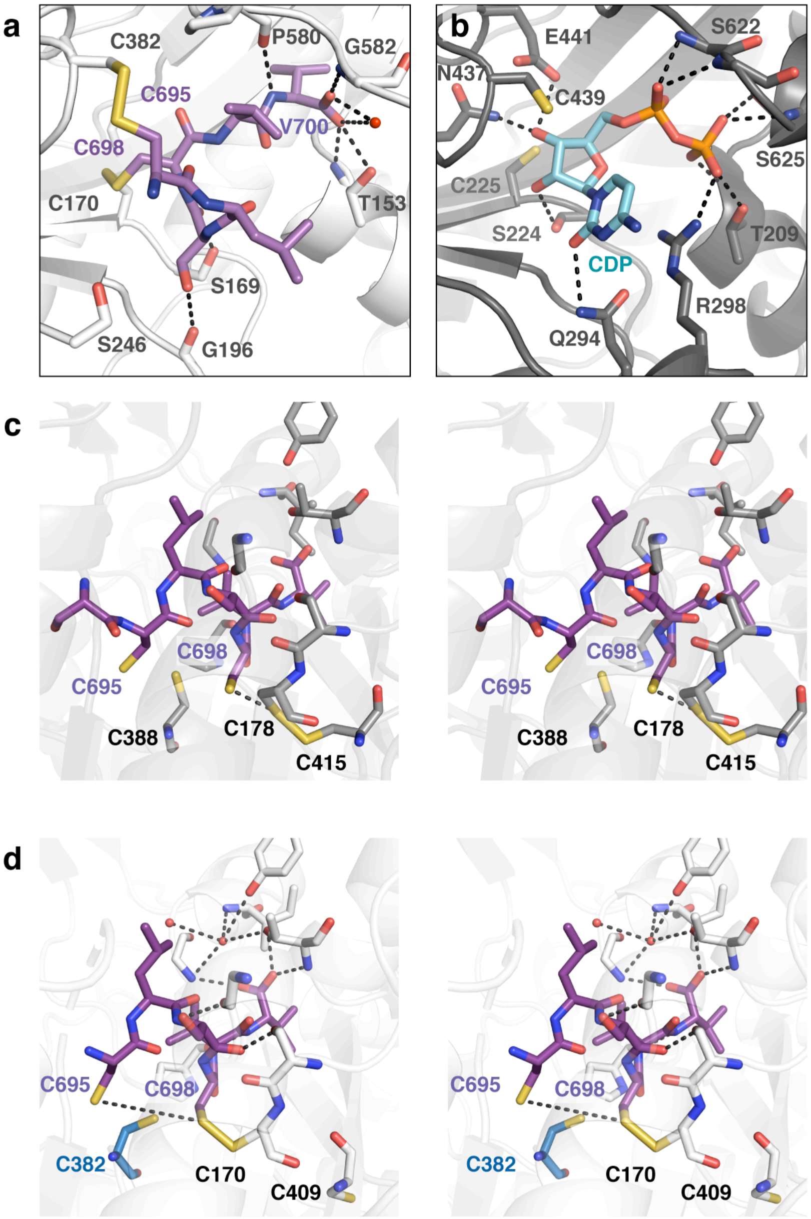

Supplementary Figure 10. Spatial arrangement of catalytically relevant cysteines. (a) The catalytic site with the disulfide-trapped C-terminus in the 2.50-Å NrdE S-dimer structure (PDB: 6MT9). The C-terminus binds in the same location as a nucleotide substrate. **(b)** The crystal structure of *E. coli* class Ia RNR shown in the same orientation as in (a) reveals polar interactions observed between the catalytic-site residues and the substrate, CDP (PDB: 5CNS)[12]. **(c)** Stereoview of the oxidized *S. typhimurium* NrdE structure (dark gray, PDB: 1PEO)[13] aligned with the C-terminus from our X-ray-reduced structure (purple sticks, PDB: 6MVE) shows C698 within 3 Å (dotted line) of the C178-C415 disulfide in the catalytic site (equivalent to C170-C409 in *B. subtilis* numbering). These three cysteines form a near-linear triad, with C698 well positioned to attack C178 (C170 in *Bs* numbering). **(d)** Stereoview of our structure with the disulfide-trapped C-terminus (white and purple sticks, PDB: 6MT9) aligned with C382 in its reduced conformation from our X-ray-reduced structure (blue sticks, PDB: 6MVE) shows that C695 is not bound in a conformation that is optimal for re-reduction. It is too distant from C698 (dotted line, 5.6 Å) to form a linear arrangement with the C698-C170 disulfide.

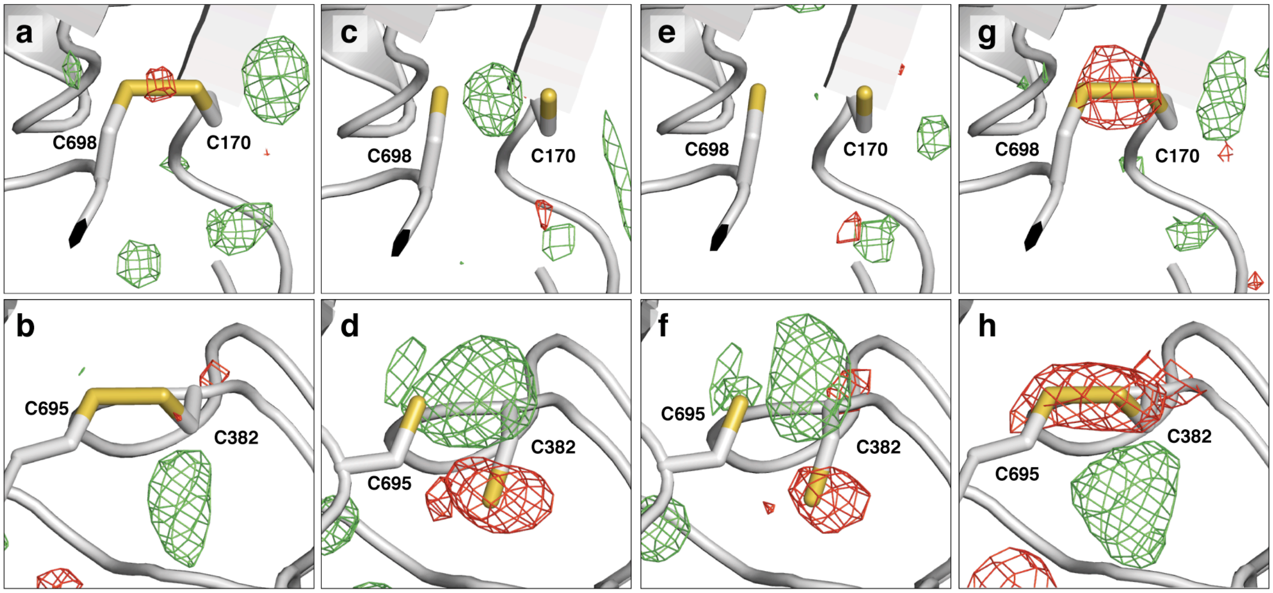

Supplementary Figure 11. Catalytic site redox state of α C-terminus trapped in crystal structures of *B. subtilis* NrdE. Both datasets show signs of partial oxidation and reduction. However, the 2.50-Å dataset is better described by an oxidized model, and the 2.55-Å dataset is better described by the reduced model. **(a-b)** mF_o-DF_c map synthesized using the refined oxidized model and 2.50-Å dataset. **(c-d)** mF_o-DF_c map synthesized using the refined reduced model and 2.50-Å dataset. **(e-f)** mF_o-DF_c map synthesized using the refined reduced model and 2.55-Å dataset. **(g-h)** mF_o-DF_c map synthesized using the refined oxidized model and 2.55-Å dataset. All maps are contoured at 3σ, with the green mesh representing positive F_o density and the red negative F_c density.

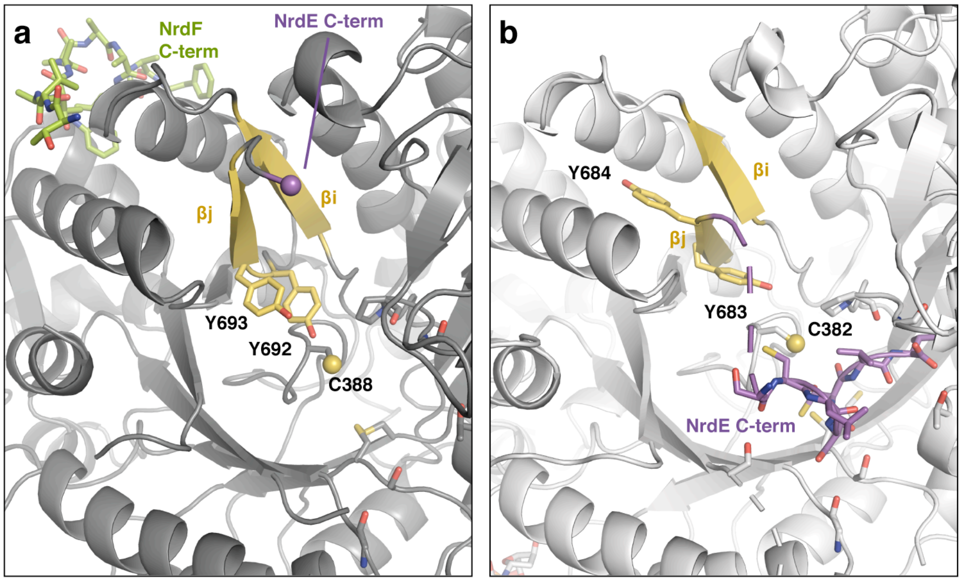

Supplementary Figure 12. Crystal structures provide insight into control of radical transfer and re-reduction (a) In the structure of the *S. typhimurium* NrdEF complex (PDB: 2BQ1)[5], the NrdE tyrosines involved in PCET (Y692/Y693) form a stacked dyad over the catalytic cysteine (C388). The tyrosine dyad is located in the β-strand (yellow) directly preceding the NrdE C-terminal tail (purple sphere). The C-terminus of NrdF (green) is observed bound within a hydrophobic cleft on the surface of the catalytic barrel in this structure. **(b)** In our structures of the NrdE catalytic site, the β-strand containing the tyrosine dyad (Y683/Y684) is partially unzipped from the β-sheet (yellow) to allow for the NrdE C-terminus (purple sticks) to reach the catalytic site (disordered region shown as purple dotted line). This change in secondary structure is accompanied by the unstacking of Y684 from Y683.

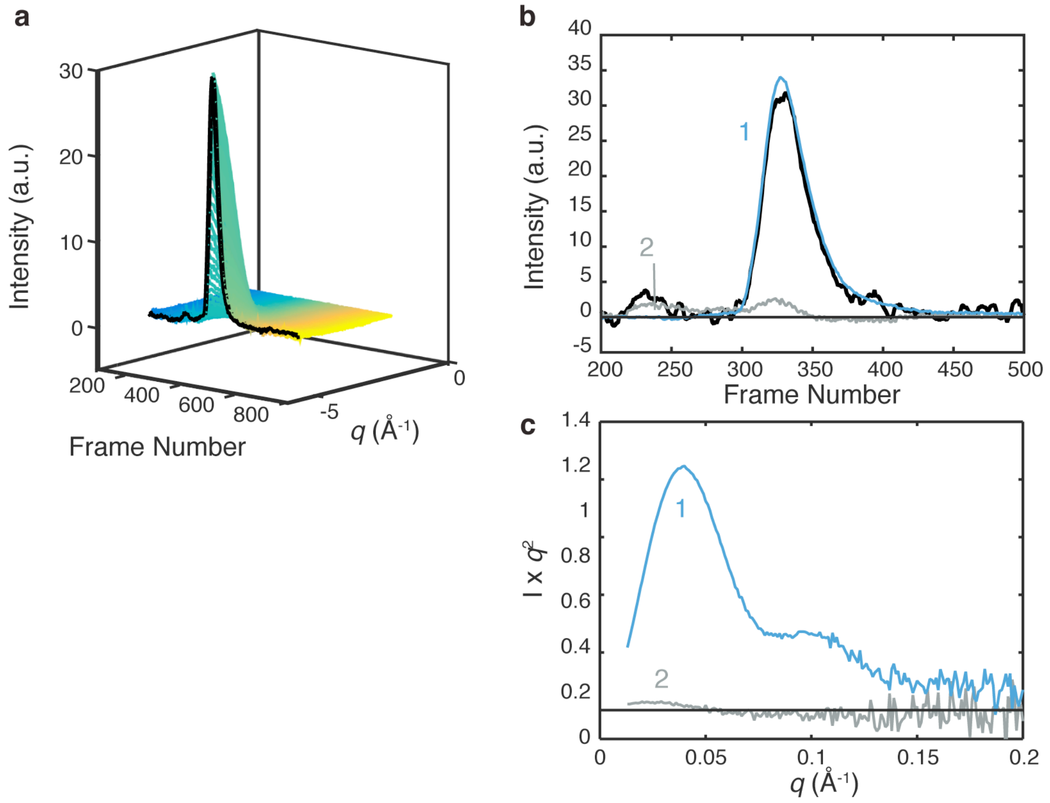

Supplementary Figure 13. NrdEF is predominantly $\alpha_2\beta_2$ under activating nucleotide conditions. (a) SEC-SAXS was performed on an equimolar solution of C382S holo-NrdE and Mn-NrdF pre-incubated with 1 mM ATP, 250 μM TTP and eluted under identical buffer conditions. **(b)** EFA of the overall elution profile (black) reveals one major protein component (1, blue) and a minor second component (2, grey). No additional protein components were observed eluting after the major component, suggesting that the two subunits bind strongly. **(c)** The Kratky plots of the two components separated by EFA. The minor component has a partially negative profile that does not resemble normal protein scattering and instead may represent the changing background scattering over the course of the elution caused by the elution of a buffer component from a previous SEC-SAXS experiment or from the accumulation of materials on the flow cell windows. Regardless, the major component has a profile with a molecular weight estimate that is consistent with an $\alpha_2\beta_2$ tetramer.

Supplemental References

1. Minnihan, E. C., Nocera, D. G. & Stubbe, J. Reversible, long-range radical transfer in E. coli class Ia ribonucleotide reductase. *Acc. Chem. Res.* **46,** 2524–2535 (2013).
2. Uhlin, U. & Eklund, H. Structure of ribonucleotide reductase protein R1. *Nature* **370,** 533–539 (1994).
3. Ando, N. *et al.* Structural interconversions modulate activity of Escherichia coli ribonucleotide reductase. *Proc. Natl. Acad. Sci.* **108,** 21046–21051 (2011).
4. Seyedsayamdost, M. R., Chan, C. T. Y., Mugnaini, V., Stubbe, J. & Bennati, M. PELDOR spectroscopy with DOPA-β2 and NH2Y-α2s: Distance measurements between residues involved in the radical propagation pathway of E. coli ribonucleotide reductase. *J. Am. Chem. Soc.* **129,** 15748–15749 (2007).
5. Uppsten, M., Färnegårdh, M., Domkin, V. & Uhlin, U. The first holocomplex structure of ribonucleotide reductase gives new insight into its mechanism of action. *J. Mol. Biol.* **359,** 365–377 (2006).
6. Kucukelbir, A., Sigworth, F. J. & Tagare, H. D. Quantifying the local resolution of cryo-EM density maps. *Nature Methods* **11,** 63–65 (2014).
7. Boal, A. K., Cotruvo, J. A., Stubbe, J. & Rosenzweig, A. C. The dimanganese(II) site of Bacillus subtilis class Ib ribonucleotide reductase. Biochemistry 51, 3861–3871 (2012).
8. Parker, M. J. *et al.* An endogenous dAMP ligand in Bacillus subtilis class Ib RNR promotes assembly of a noncanonical dimer for regulation by dATP. *Proc. Natl. Acad. Sci.* **115,** E4594–E4603 (2018).
9. Franke, D. *et al.* ATSAS 2.8: A comprehensive data analysis suite for small-angle scattering from macromolecular solutions. *J. Appl. Crystallogr.* **50,** 1212–1225 (2017).
10. Konarev, P. V., Volkov, V. V., Sokolova, A. V., Koch, M. H. J. & Svergun, D. I. PRIMUS: A Windows PC-based system for small-angle scattering data analysis. *J. Appl. Crystallogr.* **36,** 1277–1282 (2003).
11. Denessiouk, K. A., Rantanen, V. V. & Johnson, M. S. Adenine recognition: A motif present in ATP-, CoA-, NAD-, NADP-, and FAD-dependent proteins. *Proteins Struct. Funct. Genet.* **44,** 282–291 (2001).
12. Zimanyi, C. M., Chen, P. Y. T., Kang, G., Funk, M. A. & Drennan, C. L. Molecular basis for allosteric specificity regulation in class Ia ribonucleotide reductase from Escherichia coli. *Elife* **5,** e07141 (2016).
13. Uppsten, M. *et al.* Structure of the large subunit of class Ib ribonucleotide reductase from Salmonella typhimurium and its complexes with allosteric effectors. *J. Mol. Biol.* **330,** 87–97 (2003).
