# Supplementary material for: Convergent allostery in ribonucleotide reductase
Source: Nat Commun. 2019 Jun 14;10:2653. doi: 10.1038/s41467-019-10568-4 (PMC6572854; doi:10.1038/s41467-019-10568-4)
Supplement: Supplementary file 3 — Reporting Summary [file 41467_2019_10568_MOESM3_ESM.pdf]

nature research

Corresponding author(s): Nozomi Ando

Last updated by author(s): May 15, 2019

Reporting Summary

Nature Research wishes to improve the reproducibility of the work that we publish. This form provides structure for consistency and transparency in reporting. For further information on Nature Research policies, see Authors & Referees and the Editorial Policy Checklist.

Statistics

For all statistical analyses, confirm that the following items are present in the figure legend, table legend, main text, or Methods section.

n/a	Confirmed	
☒	☐	The exact sample size (n) for each experimental group/condition, given as a discrete number and unit of measurement
☒	☐	A statement on whether measurements were taken from distinct samples or whether the same sample was measured repeatedly
☒	☐	The statistical test(s) used AND whether they are one- or two-sided<br>*Only common tests should be described solely by name; describe more complex techniques in the Methods section.*
☒	☐	A description of all covariates tested
☒	☐	A description of any assumptions or corrections, such as tests of normality and adjustment for multiple comparisons
☒	☐	A full description of the statistical parameters including central tendency (e.g. means) or other basic estimates (e.g. regression coefficient) AND variation (e.g. standard deviation) or associated estimates of uncertainty (e.g. confidence intervals)
☒	☐	For null hypothesis testing, the test statistic (e.g. F, t, r) with confidence intervals, effect sizes, degrees of freedom and P value noted<br>*Give P values as exact values whenever suitable.*
☒	☐	For Bayesian analysis, information on the choice of priors and Markov chain Monte Carlo settings
☒	☐	For hierarchical and complex designs, identification of the appropriate level for tests and full reporting of outcomes
☒	☐	Estimates of effect sizes (e.g. Cohen's d, Pearson's r), indicating how they were calculated

Our web collection on statistics for biologists contains articles on many of the points above.

Software and code

Policy information about availability of computer code

Data collection	BioXTAS RAW v. 1.5.0 was used for SAXS data collection.<br>EPU v. 1.9.1.16 was used for EM data collection of the NrdEF filament.<br>SerialEM v. 3.7b12 was used for EM data collection of the NrdE filament.<br>ADX CHESS v. May 2018 was used for crystallography data collection.
Data analysis	MATLAB v. 2017b and v. 2018 were used for SAXS and sequence data a<br>nalysis using built-in toolboxes and code cited in the manuscript.<br>The ATSAS package v. 2.8.0 was used for SAXS data analysis.<br>SAXSMoW v. 2.0 was used for SAXS molecular weight estimation.<br>AllosMod-FoXS v.master.530b09f was used for SAXS structure fitting.<br><br>PARTICLE v. 1.0 was used for EM data processing and reconstruction of the NrdE filament.<br>MotionCor2 Build Nov. 14, 2016 was used to align movie frames of the NrdEF filament.<br>Gctf version 1.18 was used to fit CTF parameters of the NrdEF filament.<br>RELION version 3.0 was used for NrdEF filament processing and reconstruction.<br>cryoSparc2 version 2.0.2 and cryoSparc version 0.6.5 were also used for NrdEF filament reconstruction.<br><br>XDS v. 20180409 was used to integrate crystal diffraction images.<br>iMosflm v.7.2.2 was also used to integrate crystal diffraction images.<br>AIMLESS v. 0.7.3 was used to scale and merge diffraction data.<br>Phaser v. 2.8.2 was used to estimate phases of diffraction data.<br>Phenix v. 1.14-3260 was used to refine crystal models.<br>Coot v. 0.8.9.1 was used to manually refine crystal models.<br>MolProbity v. 4.4 was used to assess model quality.

UCSF Chimera 1.10.1 was used to visualize structure models.
Pymol v. 2.20 was also used to visualize structure models.
LigPlot+ v. 2.1 was used to visualize ligand binding in structure models.

For manuscripts utilizing custom algorithms or software that are central to the research but not yet described in published literature, software must be made available to editors/reviewers. We strongly encourage code deposition in a community repository (e.g. GitHub). See the Nature Research guidelines for submitting code & software for further information.

Data

Policy information about availability of data

All manuscripts must include a data availability statement. This statement should provide the following information, where applicable:

- Accession codes, unique identifiers, or web links for publicly available datasets
- A list of figures that have associated raw data
- A description of any restrictions on data availability

Coordinates and structure factors for crystal structures have been deposited in the Protein Data Bank under the following accession codes: disulfide-trapped S-dimer (PDB 6MT9), X-ray-reduced S-dimer (PDB 6MVE), and S-dimer with empty M-site (PDB 6MV9). EM structures and associated atomic models have been deposited in the Electron Microscopy Data Bank and the Protein Data Bank under the following accession codes: dATP-inhibited NrdEF filament (EMD-9272; PDB 6MW3) and dATP- inhibited NrdE filament (EMD-9293; PDB 6MYX). SAXS data supporting this study are available from the corresponding author upon request.

Field-specific reporting

Please select the one below that is the best fit for your research. If you are not sure, read the appropriate sections before making your selection.

☒ Life sciences ☐ Behavioural & social sciences ☐ Ecological, evolutionary & environmental sciences

For a reference copy of the document with all sections, see nature.com/documents/nr-reporting-summary-flat.pdf

Life sciences study design

All studies must disclose on these points even when the disclosure is negative.

Sample size	No population-based studies were performed in this manuscript. The only statistical analyses performed in this study are standard curve-fitting and data validation procedures in structural biology. Cryo-electron microscopy and crystallography data collection and refinement statistics are provided in Supplementary Tables 2-3. SAXS sample conditions are summarized in Supplementary Table 1. Error bars in SAXS figures are derived from curve-fitting, which are explicitly defined in the Methods.
Data exclusions	By convention, X-ray scattering and diffraction images that exhibited radiation damage (as determined by merging statistics) are excluded in the data processing step. We have no data exclusions to disclose from any of the data analyses.
Replication	Reproducibility of SAXS data was confirmed by replication of background scattering (i.e. matching before and after protein scattering) and by redundancy in the data collection (i.e. multiple frames per sample, multiple data points per experiment). These methods are detailed in Skou, et al. Nature Protocols (2014) which is cited in the manuscript.
Randomization	No experiments were performed that required randomization.
Blinding	Blinding is not relevant to our study. No experimental groups were allocated, and the nature of our research is not subject to bias that can be prevented by experimental blinding.

Reporting for specific materials, systems and methods

We require information from authors about some types of materials, experimental systems and methods used in many studies. Here, indicate whether each material, system or method listed is relevant to your study. If you are not sure if a list item applies to your research, read the appropriate section before selecting a response.

Materials & experimental systems

n/a	Involved in the study	
☒	☐	Antibodies
☒	☐	Eukaryotic cell lines
☒	☐	Palaeontology
☒	☐	Animals and other organisms
☒	☐	Human research participants
☒	☐	Clinical data

Methods

n/a	Involved in the study	
☒	☐	ChIP-seq
☒	☐	Flow cytometry
☒	☐	MRI-based neuroimaging
